# Health behaviour changes after type 2 diabetes diagnosis: Findings from the English Longitudinal Study of Ageing

**DOI:** 10.1038/s41598-018-35238-1

**Published:** 2018-11-16

**Authors:** Ruth A. Hackett, Catherine Moore, Andrew Steptoe, Camille Lassale

**Affiliations:** Department of Behavioural Science and Health, University College London, London, UK

## Abstract

Healthy lifestyle is key for type 2 diabetes (T2D) management. It is unclear whether individuals change health behaviours in response to T2D diagnosis. We compared smoking, physical activity, fruit and vegetable intake and alcohol consumption at three times (pre-diagnosis, at diagnosis, 2–4 years post-diagnosis) in individuals who developed T2D and controls. Behaviours were assessed in 6877 individuals at waves 3–7 of the English Longitudinal Study of Ageing. Generalized estimating equations were used to examine differences by group and time and group-by-time interactions. The T2D group were less active (*p* < 0.001) and consumed less alcohol (*p* < 0.001). Smoking (*p* < 0.001), alcohol consumption (*p* = 0.037) and physical activity (*p* = 0.042) decreased over time in the overall sample, fruit and vegetable intake (*p* = 0.012) and sedentary activity (*p* < 0.001) increased. A group-by-time interaction was found for smoking, with the T2D group having greater reductions in smoking over time (*p* < 0.001). No significant interactions were detected for other behaviours. We found limited evidence that T2D diagnosis encourages behaviour change, other than a reduction in smoking. Given the importance of lifestyle for T2D outcomes, strategies for motivating behaviour change need to be identified.

## Introduction

Diabetes is a chronic condition of which type 2 diabetes (T2D) is the most common form^1^. Many cases are considered preventable due to the relationship between T2D risk, adiposity and modifiable health behaviours^2^.

As well influencing T2D risk, health behaviours following T2D diagnosis are crucial to patient outcomes. Behaviours such as smoking, physical inactivity, poor diet and excessive alcohol consumption increase the risk of diabetes complications^3^, including cardiovascular disease^4^. Therefore, the importance of a healthy lifestyle is emphasized in diabetes treatment guidelines^5–7^.

The diagnosis of T2D represents a potential trigger or ‘teachable moment’^8^ for behaviour change, in which individuals become aware of the need to adopt health promoting behaviours. Trial data^9^ and data from “real world” clinical settings^10^ demonstrate that lifestyle interventions can improve behaviour, reducing T2D risk in healthy individuals. There is also data from multiple studies highlighting effective interventions for positive behaviour change in those with an existing diabetes diagnosis^11^.

Less is known about behaviour change following a T2D diagnosis on a population level, without the intensive programs used in interventional studies, and whether behaviour change differs in adults with T2D from individuals without T2D. There is evidence that levels of engagement in health protective behaviours may be similar in those with and without diabetes^12,13^, with the exception that those with T2D may be more sedentary^12,14^. However, health behaviours pre-diagnosis are likely to be unhealthier in those who go on to develop T2D. Therefore, parity with the rest of the population following diagnosis could constitute improvement.

Several longitudinal studies have investigated change in smoking behaviour at two time points, before and after T2D diagnosis. Three analyses of the US-based Health and Retirement study (HRS) found that those who develop T2D were more likely to quit smoking^15–17^. This finding was also reported in large Canadian^18^ and Australian^19^ studies. However, the one European sample to address this question did not observe any change in Dutch adults^12^. Two European studies have investigated fruit and vegetable intake following T2D diagnosis. A Swedish study of 23,953 male participants found a significant improvement in intake following diagnosis^20^. However, no change was detected in the UK-based Whitehall II cohort^21^, neither in an Australian sample^19^. Finally, the findings for change in physical activity following T2D diagnosis are also equivocal. The majority of studies report no change in activity over time, with null results reported in the HRS^16,17^, and two Australian samples^19,22^. In contrast, two studies report an improvement in physical activity following diagnosis, including an analysis of 84,300 US-based women^23^ and the large Canadian National Population Health Survey^18^. No European study has compared patterns of physical activity in healthy participants and those who develop T2D. Only two studies to date have assessed sedentary activity following T2D diagnosis, and both reported no change following diagnosis^12,23^.

Overall, the longitudinal evidence investigating behaviour change following a T2D diagnosis is mixed. Despite an increasing number of studies in the area, these have been limited by focusing on behaviour at only 2 time points^12,16–21,23^, with the exception of one analysis on physical activity alone^22^. More importantly, several of these studies failed to compare changes in behaviour over time in those with who developed T2D and a control group[13,17,19]. Finally, the majority of studies have been based in North America[16–19,24] and a full picture of health behaviour change across multiple behaviours is lacking in an English sample.

To address these limitations, the present study aimed to examine the impact of T2D diagnosis on changes in multiple health behaviours at three times (pre-diagnosis, time when diagnosis is reported, and 2–4 years post-diagnosis) in a sample of English adults.

## Methods

### Study population

The data come from the English Longitudinal Study of Ageing (ELSA); a representative study of community-dwelling English adults aged ≥50 years^24^. Data collection began in 2002–2003 (wave 1) with follow-ups biennially. A sample of 11, 393 individuals participated at wave 1. All participants gave informed consent. Ethical approval was obtained from the National Research Ethics Committee. All study methods were performed in accordance with the Helsinki Declaration and good clinical and scientific practice.

### Participants

**Figure 1:**
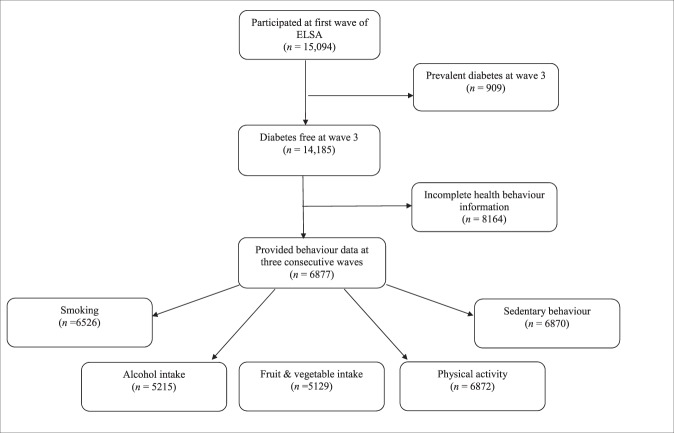
Flow diagram of participants included and excluded from the analyses. To obtain the final sample only those who were diabetes-free at wave 3 (2006–2007) and who health behaviour data at three consecutive waves for at least one behaviour of interest were included. ELSA = English Longitudinal Study of Ageing.

As fruit and vegetable intake was first evaluated at wave 3 in ELSA, this wave constitutes the study baseline. All participants who reported having T2D up to wave 3 were excluded from the present analysis. The T2D group consisted of participants (free of diabetes at wave 3) who reported a first diagnosis of T2D at either wave 4 (2008–2009), wave 5 (2010–2011) or wave 6 (2012–2013). Three time points were used in the study. We classified the wave preceding diagnosis as “pre-diagnosis” (T0), the wave when T2D was first reported as “peri-diagnosis” (T1) and the wave following diagnosis as “post-diagnosis” (T2). Individuals reporting T2D at wave 7 were excluded due to the absence of post-diagnosis data. The comparison group consisted of participants who did not receive a diagnosis in any wave. Data from waves 4, 5 and 6 were used as T0, T1 and T2 for the comparison group. For both groups, only individuals with health behaviour data at three consecutive waves for at least one behaviour of interest were included giving a final sample of 6877 participants (see Fig. 1). Of these 6877, 115 reported a new diagnosis of T2D at wave 4, 144 reported a diagnosis at wave 5 and 109 reported T2D at wave 6 giving a final sample of 368 individuals with T2D and 6509 participants who were T2D-free.

### Health behaviours

Smoking status was based on the question ‘Do you smoke cigarettes at all nowadays?’ Responses were coded as yes/no. A total of 6526 participants had smoking information at 3 time points (341 with diabetes; 6185 in the comparison group). Alcohol consumption was assessed using the item ‘On how many days out of the last seven did you have an alcoholic drink?’ Those who reported drinking on 5–7 days of the week were classified as daily alcohol drinkers. Responses were dichotomized on this basis as daily drinker (yes/no). Information on alcohol consumption was available for 5215 individuals (258 with diabetes, 4957 in the comparison group). Fruit and vegetable intake was used as an indicator of diet. At waves 3 and 4, diet was assessed with items such as ‘How much of the following did you eat yesterday’ with answers including ‘Average slices of a very large fruit, such as melon’ and ‘Salad (cereal bowlfuls)’. In waves 5–7 participants were asked ‘how many portions of fruit/vegetables do you eat on a given day’. From these items, a variable was derived to indicate whether the participant consumed ≥5 pieces of fruit or vegetables daily (yes/no). In our sample, 5129 individuals had data for this measure (254 with diabetes, 4875 in the comparison group). Physical activity and sedentary behaviour variables were derived from the question “Do you take part in any sports that are: vigorous/moderate/mildly energetic” with response options of “more than once a week/once a week/one to three times a month/hardly ever or never”. We classified individuals as “active” if they reported doing moderate or vigorous activity at least once a week and “non-active” if they reported doing less than this. We had physical activity information for 6872 participants (367 with diabetes, 6505 in the comparison group). For sedentary behaviour, participants were classified as “sedentary” if they reported ‘hardy ever or never’ participating in mild, moderate or vigorous activity. Those reporting greater levels of activity were classified as “non-sedentary”. A total of 6870 participants provided this information (367 with diabetes, 6503 in the comparison group).

### Covariates

Diabetes and health behaviours are age, sex and socially patterned^25^. Therefore, age, sex and household non-pension wealth at T0 were included as covariates. Non-pension wealth is an indicator of socio-economic status (SES) in older people^26^ and is presented in quintiles (1 = low, 5 = high) derived on the entire population. All participants had complete information on age and sex. However, 156 individuals had missing information on wealth (9 with diabetes, 147 in the comparison group).

### Statistical analysis

Demographic characteristics at pre-diagnosis (T0) were assessed using t-tests and chi-squared tests. To tabulate movement between categories (e.g. from smoker to non-smoker) from T0 to T2, McNemar’s test was computed for the groups separately. For analyses at three times, generalized estimating equation (GEE) models were used to examine main effects of group (overall group differences in the prevalence of each behaviour, independent of time), main effects of time (changes in behaviours over time, independent of group), and group-by-time interactions (differences in behaviour over time between groups). The GEE results are based on an unstructured correlation matrix. All analyses were conducted in SPSS version 24.

### Sensitivity analyses

We conducted three sets of sensitivity analyses. In the first, we added education to our models as an additional marker of SES which may influence health behaviours^27^. Three categories of education were used: no qualifications, high school certificate or A levels, university degree. Confounding by body mass index (BMI) was investigated in our second sensitivity analysis, as health behaviours and T2D are associated with obesity^28–30^. Objective BMI measurements were taken at wave 4. For the third sensitivity analysis, individuals who reported having a limiting longstanding illness at wave 4 (T0) were excluded from the comparison group. This binary variable (yes/no) was based on a self-report of any long-standing condition that participants classed as limiting their activities^31^. We conducted this analysis to compare the behaviours of the participants who developed T2D with the behaviours of a healthy comparison group, not just one with the absence of T2D.

## Results

### Descriptive characteristics

The analytic sample comprised of 6877 individuals and of these 368 developed diabetes between waves 4 and 6 (2008–2013). The characteristics of the groups at the pre-diagnosis stage (T0) are presented in Table 1. Those who developed T2D were significantly older on average (*p* = 0.033), were more likely to be male (*p* = 0.016), to have lower wealth (*p* < 0.001) and no formal qualifications (*p* < 0.001) than those in the comparison group. Individuals with T2D were more likely to report suffering from a long-term illness and coronary heart disease than people in the comparison group (*p* < 0.001). The groups did not differ in self-reported stroke or cancer (*p*’s > 0.281). Those who developed T2D had a significantly higher BMI and higher glycated haemoglobin (HbA1c) than those in the comparison group (*p’s* < 0.001).

**Table 1:** Participant characteristics at the pre-diagnosis stage for T2D and baseline for the comparison group, the English Longitudinal Study of Ageing.

	N	Diabetes group (n = 368)	N	Control group (n = 6509)	p value (T2D vs control)
		Median (IQR)		Median (IQR)	
Age (years)	368	65 (13)	6509	64 (13)	=0.033
		**N (%)**		**N (%)**	
Sex, men	368	183 (49.7%)	6509	2821 (43.3%)	=0.016
Wealth categories (%)	359		6362		<0.001
1 (lowest)		89 (24.8%)		929 (14.6%)	
2		94 (26.2%)		1151 (18.1%)	
3		72 (20.1%)		1288 (20.2%)	
4		50 (13.9%)		1432 (22.5%)	
5 (highest)		54 (15%)		1562 (24.6%)	
Education	366		6420		<0.001
No formal qualifications		120 (32.8%)		1566 (24.4%)	
High school/A-levels		205 (56%)		3565 (55.5%)	
University degree		41 (11.2%)		1289 (20.1%)	
Limiting long-standing illness (% yes)*	252	116 (46%)	4329	1334 (30.8%)	<0.001
Coronary heart disease (% yes)*	258	41 (15.9%)	4331	407 (9.4%)	<0.001
Stroke (% yes)*	258	15 (5.8%)	4331	190 (4.4%)	=0.281
Cancer (% yes)*	258	25 (9.7%)	4331	394 (9.1%)	=0.748
		**Mean (SD)**		**Mean (SD)**	
BMI (m^2^/kg)*	270	31.30 ± 5.72	5146	27.69 ± 4.73	<0.001
HbA1c (%)*	227	6.77 ± 1.14	4427	5.72 ± 0.37	<0.001

Note: The pre-diagnosis wave (T0) for the comparison group was wave 4 (2008–09).

For the diabetes group T0 could be wave 3 (2006–07), 4 (2008–09) or 5 (2010–11).

BMI = Body Mass Index; HbA1c = Glycated Haemoglobin; IQR = Interquartile range; SD = Standard deviation; T2D = Type 2 diabetes.

*These variables were reported at wave 4 for all participants.

The proportion of engagement in each lifestyle behaviour at pre- (T0) and post-diagnosis (T2) for the T2D and comparison group is presented in Table 2. In the T2D group there was a reduction in smoking (*p* = 0.041) between T0 (16.1%) and T2 (11.8%). The T2D group became more sedentary over time (7.9% at T0 and 13% at T2, *p* = 0.009). Alcohol consumption, physical activity and fruit and vegetable intake did not change significantly between T0 and T2 (*p*’s > 0.405).

**Table 2:** Health behaviour change between pre and post diagnosis waves in the diabetes and comparison groups of the English Longitudinal Study of Ageing.

Health behaviour	N	Diabetes group (n = 368)			N	Comparison group (n = 6509)		
		T0	T2	p value (T0 vs T2)		T0	T2	p value (T0 vs T2)
Smoking (% yes)	345	56 (16.1%)	43 (11.8%)	**=0**.**041**	6304	850 (13.5%)	712 (10.9%)	<0.001
Alcohol (% daily)	274	48 (15.9%)	46 (15.1%)	=0.405	5178	1413 (24.7%)	1298 (22.7%)	<0.001
Physical activity (% active)	368	58 (15.8%)	45 (12.2%)	=0.131	6508	2089 (32.1%)	1885 (29%)	<0.001
Sedentary (% yes)	368	29 (7.9%)	48 (13%)	**=0**.**009**	6507	327 (5%)	432 (6.6%)	<0.001
≥5 portions fruit & vegetables daily (% yes)	269	153 (50.3%)	168 (56.4%)	=0.156	5123	3271 (57.3%)	3386 (59.9%)	<0.001

Note: T0 is the pre-diagnosis wave and T2 is the post-diagnosis wave.

The comparison group represents the trend of behaviour change in older adults without T2D. In this group, there was a significant change in all behaviours between T0 and T2. The proportion of smokers fell and there were corresponding decreases in alcohol consumption and physical activity, alongside with an increase in sedentary behaviour (*p*’s < 0.001). The number of people who ate ≥5 portions of fruit and vegetables daily increased over time (*p* < 0.001).

### Smoking

**Figure 2:**
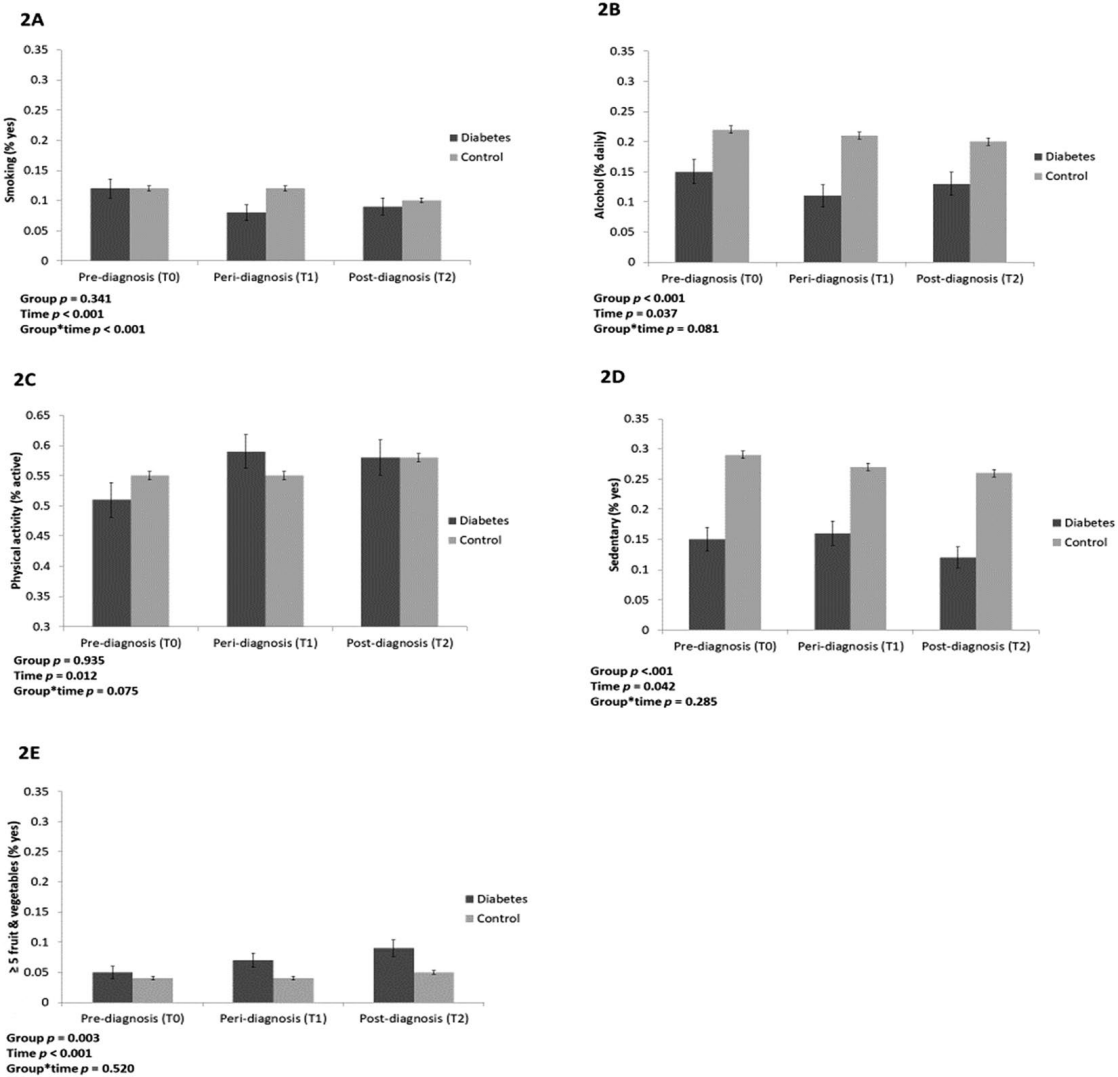
Health behaviours in the diabetes and control groups. (**A**) Proportion of smokers. (**B**) Proportion of daily alcohol drinkers (drinking 5 days a week or more). (**E**) Proportion eating five or more portions of fruit and vegetables daily. (**C**) Proportion engaging in weekly moderate or vigorous activity. (**D**) Proportion engaging in sendentary behaviour. Proportion of participants engaging with each behaviour in the diabetes and comparison group at three time points. All proportions are adjusted for age, sex and wealth.

The adjusted proportion of smokers at each time point in the two groups is presented in Fig. 2A. There was a significant main effect of time (*p* < 0.001), with the proportion of smokers decreasing over time, independent of group. The main effect of group was not significant (*p* = 0.341), suggesting that the proportion of smokers did not differ overall between the groups, independent of time. There was a significant group-by-time interaction (*p* < 0.001) with an overall bigger drop in proportion of smokers in the T2D group than in the comparison group. In the T2D group, the proportion of smokers decreased sharply at time of diagnosis but increased slightly after diagnosis.

### Alcohol

Figure 2B shows the adjusted proportion of daily alcohol drinkers over time. There was a significant group difference in alcohol consumption, independent of time (*p* < 0.001), age, sex and wealth, with consistently lower levels of alcohol consumption in the T2D group. There was a significant main effect of time (*p* = 0.037), with the proportion of participants consuming alcohol on a daily basis decreasing over time in both groups. From T0 to T1, alcohol consumption fell from 15% to 11% in the T2D group and from 22% to 21% in the comparison group. Between T1 and T2 consumption increased in the T2D group (11% to 13%) and decreased in the comparison group (21% to 20%). However, the group-by-time interaction did not reach significance (*p* = 0.081).

### Physical activity

The results for engagement in weekly moderate or vigorous physical activity are presented in Fig. 2C. There was a significant main effect of group (*p* < 0.001), with consistently lower levels of activity in the T2D than the comparison group. There was a significant main effect of time (*p* = 0.042), with levels generally decreasing over the 3 time points. Between T0 and T1, the proportion of those engaged in physical activity slightly increased in the T2D group from 15% to 16%, while levels fell in the comparison group (29% to 27%). From T1 to T2 physical activity decreased in both groups, from 16% to 12% in the T2D group and from 27% to 26% in the comparison group. There was no significant group-by-time interaction for physical activity (*p* = 0.285).

### Sedentary behaviour

The proportion of sedentary participants at each time point can be found in Fig. 2D. A larger proportion of the T2D group were sedentary (significant main effect of group, *p* = 0.003) and the both groups showed an increase in sedentary behaviour over time (p < 0.001). In the T2D group, the proportion increased steadily from 5% (T0) to 9% (T2) whereas in the comparison group, the percentage stayed constant first (4% at T0 and T1) then increased slightly to 5%. The group-by-time interaction was non-significant (*p* = 0.520).

### Fruit and vegetables

The adjusted proportion of those consuming at least 5 fruit and vegetables a day can be found in Fig. 2E. There was a significant main effect of time (*p* = 0.012) and borderline significant group-by-time interaction (*p* = 0.075). The proportion of those in the T2D group eating ≥5 portions daily increased following diagnosis between T0 and T1 (51% to 59%). There was no corresponding change in the comparison group (55% at T0 and T1). Between T1 and T2 the proportion of those getting the recommended intake fell slightly in the T2D group (59% to 58%), whereas consumption increased in the comparison group (55% to 58%), meaning that at T2 the same proportion had an adequate fruit and vegetable intake in both groups. Accordingly, overall group difference was non-significant (*p* = 0.935).

### Sensitivity analyses

In our first set of sensitivity analyses we added education to the models. The pattern of results did not change for any behaviour: there was a decrease in smoking in the T2D group over time that differed significantly from the control group (*p*-interaction < 0.001), but no significant interactions over time for the other health behaviours.

We tested whether adding BMI altered our results (n = 5416). Overall the results were unchanged for smoking and fruit and vegetables intake. The change in alcohol consumption and in physical activity over time became non-significant, and the group difference in sedentary behaviour was slightly attenuated (*p* = 0.077), all likely due to sample size reduction.

For our final sensitivity analysis, we excluded participants in the comparison group who reported a limiting long-term illness at T0. The pattern of results was unchanged. The significant group-by-time interaction (*p* < 0.001) for smoking remained. For all other measures the group-by-time interactions did not reach significance.

## Discussion

In this sample of community-dwelling English adults, we found limited evidence that T2D diagnosis encourages positive health behaviour change, other than a reduction in smoking. Participants with T2D and controls did not differ on average in smoking. The T2D group were less likely to be physically active and to consume alcohol than the controls. They were more likely to be sedentary. No significant changes in alcohol consumption, physical activity or sedentary activity following diagnosis were detected compared to a control group free of T2D. The groups did not differ in fruit and vegetable consumption on average and no significant change in intake was detected following T2D diagnosis. There was a general reduction in smoking, alcohol consumption and fruit and vegetable intake but an increase in sedentary behaviour and decrease in physical activity engagement over the study period. However, these changes were unrelated to T2D, except for smoking.

Our finding that smoking rates fall following T2D diagnosis is in line with the majority of previous studies that have looked at change in smoking behaviour at two time points^15–19^. This result adds to the literature by demonstrating this positive change in health behaviour in an English sample for the first time and showing that this change is maintained 2–4 years following a diagnosis.

We found that smoking rates fell over time, independent of group. This is in line with trajectories in the UK, whereby smoking rates have been falling since the 1970s^32^. Our findings suggest that T2D diagnosis is an impetus to quit smoking beyond population trends. This is encouraging as smoking is a well-established risk factor for many chronic diseases including cardiovascular disease, the leading cause of death in those with diabetes^33^.

We observed no significant group-by-time interaction for alcohol consumption, suggesting that the diagnosis of T2D does not spur a reduction in intake. However, those with T2D consumed less alcohol on average than the comparison group, which is in line with other data from the UK suggesting that individuals with chronic disease consume less alcohol^34^. Our findings do not support the notion of a ‘sick quitter effect’^34^, as lowered consumption was not observed in response to a diagnosis. Data from other studies on alcohol intake post-diagnosis of T2D suggest that changes tend to vary based on the level of initial consumption. An improvement in excessive alcohol intake^16–18^ and no change in average consumption has been consistently reported^12,16^. Our measure of alcohol intake classified individuals as daily drinkers if they reported consuming alcohol 5–7 days per week. This measure is limited as we are unable to ascertain the number of units of alcohol consumed. Light drinkers consuming 1–2 units daily would still fall within the UK recommended intake guidelines^35^. Therefore, we did not tease out differences between excessive and moderate alcohol consumption in this study.

Previous research on fruit and vegetable intake following T2D diagnosis has produced inconsistent results. We observed no significant change in the proportion of individuals consuming 5 or more portions of fruit and vegetables a day following diagnosis. This is in agreement with earlier findings from another English cohort^21^ and an Australian sample of 54,858 individuals^19^. In contrast, a study of Swedish men detected an improvement in intake following diagnosis^20^. It is possible that the lack of female participants in this sample could have contributed to these diverging results. Men are reported to have less healthy dietary patterns on average than women and therefore they may have more room for improvement^36^. These findings are disappointing as nutrition is an integral part of diabetes management^5^, with fruit and vegetable intake emphasized for weight control and low glycaemic load.

We observed no significant change in physical activity in response to diagnosis in keeping with earlier findings from the HRS^16,17^, and two Australian samples^19,22^. Other studies have reported some improvements in activity following diagnosis. In a large sample of US-based women^23^ total active hours a week increased in those who developed diabetes, but no change in mild, moderate or vigorous activity was reported. A Canadian cohort of both men and women^18^ found that leisure physical activity increased following diagnosis. However, this study lacked a comparison sample and the authors suggest that it was younger participants (aged 50–64 years) who were driving the effect. The mean age in the current study was 66.17 years, but null associations have been reported in other studies with average ages of less than 56 years^16,22^ so it is unlikely age differences account for the diverging results. The T2D group in the current study were less likely to engage in physical activity than the controls. These low levels of initial activity may have acted as a barrier to change in our sample, though they do offer scope for improvement in terms of future interventional work.

The participants with T2D were more likely to be sedentary than the comparison group, in agreement with earlier work^12,14^. We did not observe an improvement in sedentary behaviour, defined as lack of activity in any category, in the present analysis, but rather the opposite with a clear increase over time. This is in line with previous studies in the area which looked at sedentary behaviour at two time points^12,23^.

Competing theoretical models on health behaviour change provide possible insights into why individuals fail or succeed at adhering to treatment or lifestyle guidelines following a diagnosis of T2D. Models of behaviour change suggest that awareness of a health problem and saliency of susceptibility to complications^37,38^ could encourage a behavioural shift. Additionally, awareness of the benefits of transitioning to a healthy lifestyle for disease prognosis^37^ and potential social expectations of change post-diagnosis have been theorized to encourage change^37,39^. However, barriers to change are also acknowledged in these theories^37^ and as unhealthy behaviours are risk factors for T2D^2^, it is likely that these behaviours are habitual and difficult to change for many individuals^40^. However, we had no information on factors related to psychological adaptation to T2D in this sample.

The present study used data from a representative English cohort. To our knowledge, it is the first to assess multiple health behaviours at pre-diagnosis, report of diagnosis and 2–4 years post-diagnosis in individuals who developed T2D and in a comparison group. The use of 3 times is important, as transient changes in initial response to diagnosis, as well as changes 2–4 year later are captured. However, reports of T2D were self-reported which makes it possible that we missed some cases, though evidence suggests there is good agreement between self-reported and physician registered diabetes diagnoses^41,42^. All behaviours were self-reported meaning that social desirability bias may have led to an underestimation of negative health behaviour. Additionally, the diet and alcohol measures varied over the study period, which may have compromised the reliability of comparing behaviours across waves. We had no information on whether participants received advice or education on health behaviours from a health professional following T2D diagnosis in line with treatment guidelines^5^. Additionally, we were unaware whether the participants with T2D had been prescribed a certain type of medication or a particular diet for their condition. The use of a comparison sample helped disentangle the potential effect of diagnosis of T2D, irrespective of temporal trend or change in the methodology. Moreover, the exact date of T2D diagnosis was unknown and happened between T0 and T1, 2 years apart. Therefore the “peri diagnosis” T1 behaviour measurement was not exactly at the same moment as diabetes diagnosis but 0 to 2 years after diagnosis. Similarly, T2 was 2 to 4 years after diagnosis. To examine changes over multiple time points, our analyses were restricted to cases with three consecutive waves of data, reducing the sample size and potentially inducing selection bias. We included participants with three waves of data for at least one health behaviour of interest. However, our study was limited by missing data meaning that for each behaviour assessed the sample used differed. Finally, while we included covariates in our analyses and conducted sensitivity checks, controlling for demographic and health-related variables does not tease out the complexities of the relationship between these and T2D. While beyond the remit of this study, an exploration of these relationships would shed light on the motivations behind positive or negative behaviour change.

Overall, we found limited evidence that T2D diagnosis in older adults is a ‘teachable moment’ for health behaviour change, other than smoking. Further research is required to understand how to motivate and facilitate behaviour change in community-dwelling populations with T2D.

## References

[1] International Diabetes Federation. *IDF Diabetes Atlas*. (International Diabetes Federation, 2015).

[2] Ley SH (2016). Contribution of the Nurses’ Health Studies to Uncovering Risk Factors for Type 2 Diabetes: Diet, Lifestyle, Biomarkers, and Genetics. Am. J. Public Health.

[3] Fowler MJ (2008). Microvascular and Macrovascular Complications of Diabetes. Clin. Diabetes.

[4] Yusuf S (2004). Effect of potentially modifiable risk factors associated with myocardial infarction in 52 countries (the INTERHEART study): case-control study. Lancet.

[5] American Diabetes Association (2018). 4. Lifestyle Management: Standards of Medical Care in Diabetes—2018. Diabetes Care.

[6] Rydén L (2014). ESC Guidelines on diabetes, pre-diabetes, and cardiovascular diseases developed in collaboration with the EASD. Eur. Heart J..

[7] Newman JD, Schwartzbard AZ, Weintraub HS, Goldberg IJ, Berger JS (2017). Primary Prevention of Cardiovascular Disease in Diabetes Mellitus. J. Am. Coll. Cardiol..

[8] McBride CM, Emmons KM, Lipkus IM (2003). Understanding the potential of teachable moments: the case of smoking cessation. Health Educ. Res..

[9] Look AHEAD Research Group *et al*. Cardiovascular effects of intensive lifestyle intervention in type 2 diabetes. *N*. *Engl*. *J*. *Med*. **369**, 145–154 (2013). 10.1056/NEJMoa1212914 PMC3791615 23796131

[10] Dunkley AJ (2014). Diabetes prevention in the real world: effectiveness of pragmatic lifestyle interventions for the prevention of type 2 diabetes and of the impact of adherence to guideline recommendations: a systematic review and meta-analysis. Diabetes Care.

[11] Fox CS (2015). Update on Prevention of Cardiovascular Disease in Adults With Type 2 Diabetes Mellitus in Light of Recent Evidence: A Scientific Statement From the American Heart Association and the American Diabetes Association. Diabetes Care.

[12] van Gool CH, Kempen GIJM, Penninx BWJH, Deeg DJH, van Eijk JTM (2007). Chronic disease and lifestyle transitions: results from the Longitudinal Aging Study Amsterdam. J. Aging Health.

[13] King DE, Mainous AG, Carnemolla M, Everett CJ (2009). Adherence to healthy lifestyle habits in US adults, 1988-2006. Am. J. Med..

[14] Hamer M, Bostock S, Hackett R, Steptoe A (2013). Objectively assessed sedentary time and type 2 diabetes mellitus: a case–control study. Diabetologia.

[15] Keenan PS (2009). Smoking and Weight Change After New Health Diagnoses in Older Adults. Arch. Intern. Med..

[16] Newsom JT (2012). Health Behavior Change Following Chronic Illness in Middle and LaterLife. J. Gerontol. B. Psychol. Sci. Soc. Sci..

[17] Xiang X (2016). Chronic Disease Diagnosis as a Teachable Moment for Health Behavior Changes Among Middle-Aged and Older Adults. J. Aging Health.

[18] Newsom JT (2012). Health behaviour changes after diagnosis of chronic illness among Canadians aged 50 or older. Health Rep..

[19] Chong S (2017). Lifestyle Changes After a Diagnosis of Type 2 Diabetes. Diabetes Spectr..

[20] Olofsson C (2017). Changes in fruit, vegetable and juice consumption after the diagnosis of type 2 diabetes: a prospective study in men. Br. J. Nutr..

[21] Akbaraly TN (2016). Little Change in Diet After Onset of Type 2 Diabetes, Metabolic Syndrome, and Obesity in Middle-Aged Adults: 11-Year Follow-up Study. Diabetes Care.

[22] Dontje ML (2016). Effect of diagnosis with a chronic disease on physical activity behavior in middle-aged women. Prev. Med..

[23] Schneider KL (2014). Change in physical activity after a diabetes diagnosis: opportunity for intervention. Med. Sci. Sports Exerc..

[24] Steptoe A, Breeze E, Banks J, Nazroo J (2013). Cohort profile: the English Longitudinal Study of Ageing. Int J Epidemiol.

[25] Imkampe AK, Gulliford MC (2011). Increasing socio-economic inequality in type 2 diabetes prevalence—Repeated cross-sectional surveys in England 1994–2006. Eur. J. Public Health.

[26] Banks, J., Karlsen, S. & Oldfield, Z. Socio-economic position. In *Health*, *wealth and lifestyles of the older population in England* 71–125 (Institute forFiscal Studies 2003).

[27] Baker DP, Leon J, Smith Greenaway EG, Collins J, Movit M (2011). The Education Effect on Population Health: A Reassessment. Popul. Dev. Rev..

[28] Hills AP (2010). Resistance training for obese, type 2 diabetic adults: a review of the evidence. Obes. Rev. Off. J. Int. Assoc. Study Obes..

[29] Ledoux TA, Hingle MD, Baranowski T (2011). Relationship of fruit and vegetable intake with adiposity: a systematic review. Obes. Rev. Off. J. Int. Assoc. Study Obes..

[30] Collaborators TG (2017). 2015 O. Health Effects of Overweight and Obesity in 195 Countries over 25 Years. N. Engl. J. Med..

[31] McMunn, A., Hyde, M., Janevic, M. & Kumari, M. Chapter 6: Health. In *Health*, *wealth and lifestyles of the older population in England: The 2002 English Longitudinal Study of Ageing* 207–230 (Institute forFiscal Studies, 2003).

[32] Office for National Statistics. Adult smoking habits in the UK: 2016. Available at, https://www.ons.gov.uk/peoplepopulationandcommunity/healthandsocialcare/healthandlifeexpectancies/bulletins/adultsmokinghabitsingreatbritain/2016 (2017).

[33] Risk E (2011). Factors Collaboration. Diabetes Mellitus, Fasting Glucose, and Risk of Cause-Specific Death. N. Engl. J. Med..

[34] Ng Fat L, Cable N, Shelton N (2015). Worsening of Health and a Cessation or Reduction in Alcohol Consumption to Special Occasion Drinking Across Three Decades of the Life Course. Alcohol. Clin. Exp. Res..

[35] Department of Health. *UK Chief Medical Officers’ Alcohol Guidelines Review Summary of the proposed new guidelines*. (Williams Lea, 2015).

[36] Roberts Katharine, Cade Janet, Dawson Jeremy, Holdsworth Michelle (2018). Empirically Derived Dietary Patterns in UK Adults Are Associated with Sociodemographic Characteristics, Lifestyle, and Diet Quality. Nutrients.

[37] Rosenstock IM (1974). Historical Origins of the Health Belief Model. Health Educ. Monogr..

[38] Prochaska, J. O. & DiClemente, C. C. Toward a comprehensive model of change. In *Treating addictive behaviors* 3–27 (Springer, 1986).

[39] Ajzen I (1991). The theory of planned behavior. Organ. Behav. Hum. Decis. Process..

[40] Rushforth B, McCrorie C, Glidewell L, Midgley E, Foy R (2016). Barriers to effective management of type 2 diabetes in primary care: qualitative systematic review. Br. J. Gen. Pract. J. R. Coll. Gen. Pract..

[41] Pastorino S (2015). Validation of self-reported diagnosis of diabetes in the 1946 British birth cohort. Prim. Care Diabetes.

[42] Smith B (2008). Challenges of self-reported medical conditions and electronic medical records among members of a large military cohort. BMC Med. Res. Methodol..

